# Restored expression of vitamin D receptor and sensitivity to 1,25-dihydroxyvitamin D_3_ in response to disrupted fusion *FOP2*–*FGFR1* gene in acute myeloid leukemia cells

**DOI:** 10.1186/s13578-016-0075-9

**Published:** 2016-02-02

**Authors:** Aleksandra Marchwicka, Aoife Corcoran, Klaudia Berkowska, Ewa Marcinkowska

**Affiliations:** Laboratory of Protein Biochemistry, Faculty of Biotechnology, University of Wroclaw, Joliot-Curie 14a, 50-383 Wroclaw, Poland

**Keywords:** DNA delivery, Leukemia, Differentiation, Vitamin D receptor, Fusion protein, Transcription factor, Interferon signaling

## Abstract

**Background:**

Acute myeloid leukemia (AML) cells can be induced to undergo terminal differentiation with subsequent loss of tumorigenicity using 1,25-dihydroxyvitamin D_3_ (1,25D) alone or in combination with hematopoietic cytokines. KG1 cells are resistant to 1,25D-induced cell differentiation. These cells have the aberrant signal transduction resulting from a constitutively active fusion protein FOP2-FGFR1, a constitutively active STAT1 and a high level of interferon (IFN) stimulated genes (ISGs).

**Methods:**

In this paper we report that in KG1 cells with constitutively activated protein FOP2-FGFR1 delivery of plasmid DNA disrupted *FOP2-FGFR1* fusion gene.

**Results:**

As a consequence, STAT1 signal transduction pathway became switched off, the expression of vitamin D receptor (VDR) gene was increased and sensitivity to 1,25D-induced differentiation was restored. The activation of ISGs in KG1 cells resulted in resistance to externally added IFNs, and also this effect was reversed in cells with disrupted *FOP2-FGFR1* fusion gene.

**Discussion:**

In this paper we have documented for the first time a link between constitutively active STAT1 signal transduction pathway, high level of ISGs and low expression of *VDR* gene.

**Conclusions:**

We show in this paper that delivery of plasmid DNA to the cells may disrupt fusion gene *FOP2-FGFR1* which occurs in a disease entity called 8p11 myeloproliferative syndrome. Inhibition of the FOP2-FGFR1 signal transduction pathway restored sensitivity of the cells to 1,25D-induced cell differentiation.

## Background

Chromosomal translocations are characteristic features of lymphoma and leukemia. A number of malignancies are driven by chromosomal translocations which involve the gene for fibroblast growth factor receptor 1 (*FGFR1*) and fuse it to the distant aminoterminal partners. In blood cells, these translocations are associated with the disease entity called 8p11 myeloproliferative syndrome, which rapidly transforms to acute myeloid leukemia (AML) [1]. The only available cell line model for this disease is the KG1 cell line, where FGFR1 oncogene partner 2 (*FOP2*)–*FGFR1* fusion gene was identified, which results in the generation of a constitutively active fusion protein FOP2–FGFR1 [2]. KG1 cells have been characterized by a constitutive activation of signal transducer and activator of transcription (STAT) 5 [2] and STAT1 [3]. Under physiological conditions interferons (IFNs) activate STAT signal transduction pathways, leading to transcription of IFN-stimulated genes (ISGs) [4]. This is the basic immune mechanism which controls the spread of viral infections. OAS proteins which activate degradation of viral RNA by 2′,5′-oligoadenylate-dependent ribonuclease L (RNAse L) are among ISGs [5, 6]. Other ISGs include the one that encodes protein MX1, which inhibits the replication cycle of influenza virus [7]. *G1P2* encodes a ubiquitin-like protein which binds to target proteins in response to IFNα or IFNβ stimulation and has chemotactic activity of neutrophils [8], while *IFIT1* gene encodes a protein which may inhibit viral replication and translational initiation [9].

AML is characterized by the accumulation of primitive hematopoietic blast cells, which lose their ability of normal differentiation [10]. AML cells can be induced to undergo terminal differentiation with subsequent loss of tumorigenicity. However, at present the clinical success of differentiation therapy for AML is limited to one rare subtype, which can be cured using *all*-trans retinoic acid (ATRA) [11]. There is a need to develop differentiation therapies to other subtypes, for example, using 1,25-dihydroxyvitamin D_3_ (1,25D) alone or in combination with hematopoietic cytokines or phytonutrients [12]. KG1 cells have been reported to be resistant to 1,25D-induced differentiation [13], and our earlier experiments revealed that this was caused by a very low expression level of vitamin D receptor (VDR) gene and protein [14]. There are hundreds of VDR-controlled genes, many of them responsible for maintaining the calcium-phosphate homeostasis [15], however, there are also many involved in blood cell functions, exemplified by CD14, a macrophage co-receptor for bacterial LPS [16]. VDR is not essential for blood cells development, but is important for their proper function [17, 18], thus low VDR level and low VDR activity in leukemic cells may contribute to their malignant phenotype.

In this study we have addressed the possible reasons of KG1 cells’ resistance to 1,25D-induced differentiation. In our search for the role of interactions between various nuclear receptors, we wanted to generate genetically modified KG1 subline with retinoic acid receptor α (*RARA*) gene silenced. Using electroporation DNA delivery method we have obtained two sublines: KG1-CtrA (transfected with a plasmid containing scrambled DNA sequence) and KG1-RARA (transfected with the plasmid coding short hairpin (sh) RNA against *RARA* gene). In both transfected cell lines VDR gene and protein expression levels increased and 1,25D-resistance was reversed, however this was not due to the gene silencing. We have therefore addressed the molecular events that have led to the reversal of 1,25D resistance. We found that the high level of *FOP2*–*FGFR1* and ISGs transcription, constitutively present in KG1 cells, were suppressed in KG1-CtrA and KG1-RARA cells. Similarly, constitutive activity of STAT1 in KG1 cells, was not longer present in transfected cells. In contrast, in KG1-CtrA and KG1-RARA cells the expression and activity of VDR were much higher than in KG1 cells. The high activation of ISGs in KG1 cells resulted in resistance to externally added IFNs, and also this effect was reversed in transfected cells. The low level of *VDR* expression in KG1 cells wasn’t caused by the repressed transcription, but at least in part by degradation of *VDR* mRNA. Addition of curcumin, an inhibitor of RNAse L, to KG1 cells partly restored 1,25D-induced cell differentiation.

## Results

### Differentiation of KG1, HL60, KG1-CtrA and KG1-RARA

**Fig. 1:**
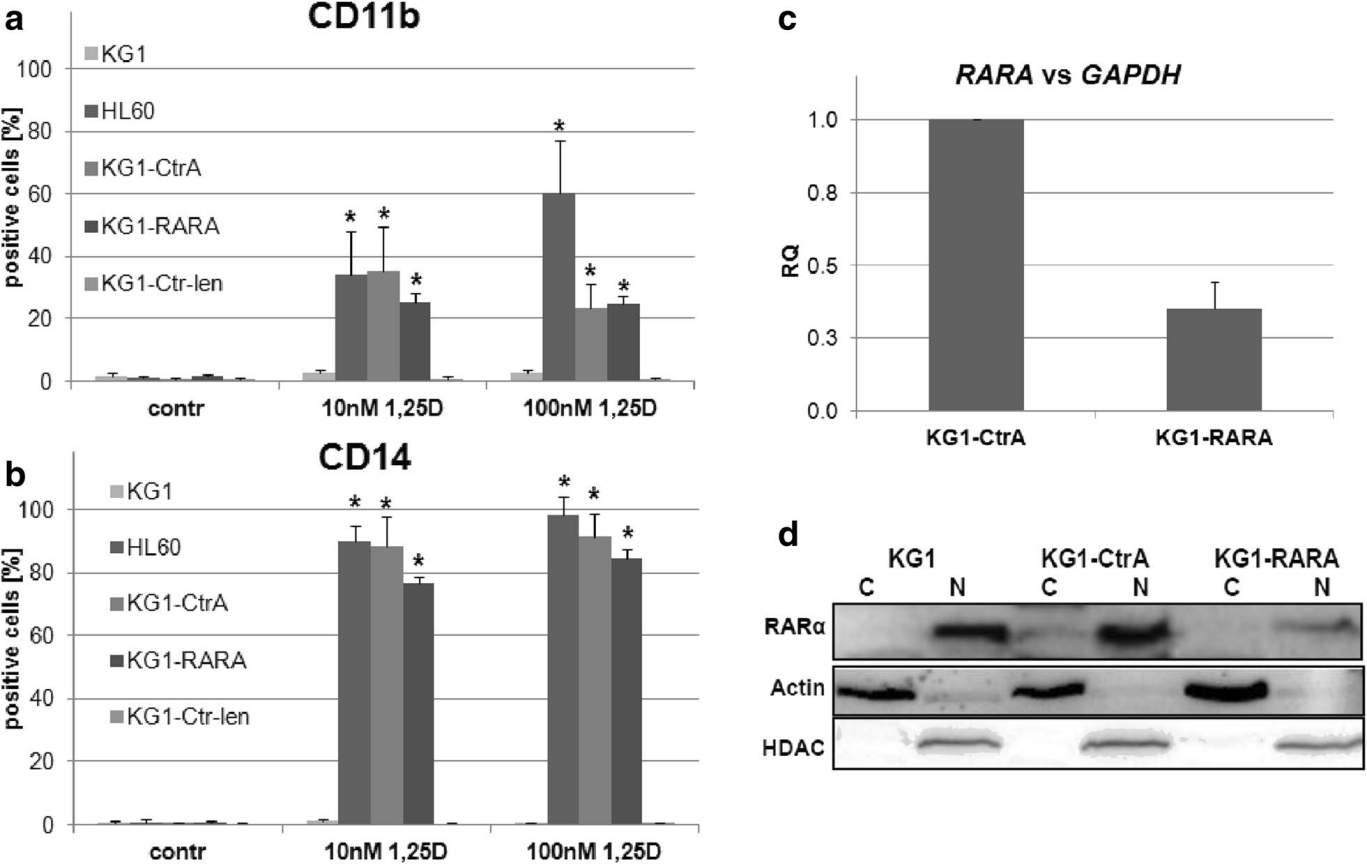
Differentiation of AML cell lines in response to 1,25D. KG1, HL60, KG1-CtrA, KG1-RARA and KG1-Ctr-len cells were exposed to 1,25D (10 or 100 nM) for 96 h and then the expression of CD11b (**a**) and CD14 (**b**) differentiation markers was detected using flow cytometry. The experiments were repeated 3–4 times and the mean percentages of positive cells (±SEM) are presented in Y-axis. The samples that differ significantly from the control are marked with *asterisk* (p < 0.05). To verify *RARA* gene silencing in KG1-RARA cells, the expression levels of *RARA* mRNA (**c**) in KG1-CtrA and KG1-RARA cell lines were measured by Real-time PCR relative to *GAPDH* expression levels. The *bar charts* show mean values (±SEM) of relative quantity (RQ). The levels of RARα protein were determined in the cytosol and nuclei of KG1, KG1-CtrA and KG1-RARA cells by western blots (**d**). The cytosolic (C) and nuclear (N) extracts were separated by SDS-PAGE, transferred to PVDF membranes and the proteins were revealed using anti-RARα, anti-actin and anti-HDAC antibodies

There are many AML cell lines available, which have variable susceptibilities to 1,25D-induced differentiation [19]. Usually the cell differentiation is tested by measuring levels of CD11b and CD14 cell surface proteins. CD11b is a cell adhesion molecule present mostly on the surface of granulocytes and monocytes [20], while CD14 is a co-receptor for bacterial lipopolysaccharide characteristic for monocytes and macrophages [21]. HL60 cell line responded to 1,25D with upregulation of CD11b and CD14 cell differentiation markers, while KG1 cells were unresponsive [14]. In a search of molecular reasons we decided to transfect KG1 cells with plasmids which encode shRNA against *RARA*. In order to properly validate our experiment we also transfected the cells with a control plasmid (CtrA), which codes scrambled sequence of shRNA. This way we obtained KG1-CtrA and KG1-RARA sublines of KG1 cells. Then the wild-type and transfected cells were exposed to 1,25D for 96 h and tested for cell differentiation markers in flow cytometry. HL60 cells which are sensitive to 1,25D moderately increased the expression of CD11b (Fig. 1a), and strongly increased CD14 (Fig. 1b). In KG1 cells the cell surface markers remained at the control levels, while KG1-CtrA cells increased the expression of CD14 similarly to HL60 cells. In KG1-RARA cells exposed to 1,25D the expression of CD14 was comparable to KG1-CtrA cells. It should be noted that when HL60 cells were transfected with CtrA plasmid, their 1,25D-induced differentiation was similar to that of wild type HL60 cells [22], and when KG1 cells were transformed using lentiviral vector encoding scrambled shRNA they remained resistant to 1,25D (KG1-Ctr-len cells in Fig. 1a, b).

In order to validate whether the expression of *RARA* gene was indeed efficiently knocked down in KG1-RARA cells, the RARα mRNA (Fig. 1c) and protein levels (Fig. 1d) were compared in KG1-CtrA and KG1-RARA cells. The mRNA expression was reduced to approximately 40 % of initial level, and was followed by reduced RARα protein content in the nuclei of KG1-RARA cells.

The plasmids that were used in our experiments confer the resistance to puromycin, an antibiotic which is toxic to eukaryotic cells. Transfected KG1 cells were selected from untransfected in the culture using this antibiotic. Since puromycin inhibits protein translation, it seemed unlikely that the effect of 1,25D-induced differentiation was caused by the exposure of the cells to puromycin. However, in order to verify that, we cultured KG1 cells at sub-lethal concentrations of puromycin (250 nM) and exposed them to 10 nM 1,25D and we detected that KG1 still did not differentiate (not shown). These experiments confirmed that cell differentiation of KG1-CtrA and KG1-RARA cells wasn’t caused by puromycin.

### VDR in HL60, KG1, KG1-CtrA and KG1-RARA

**Fig. 2:**
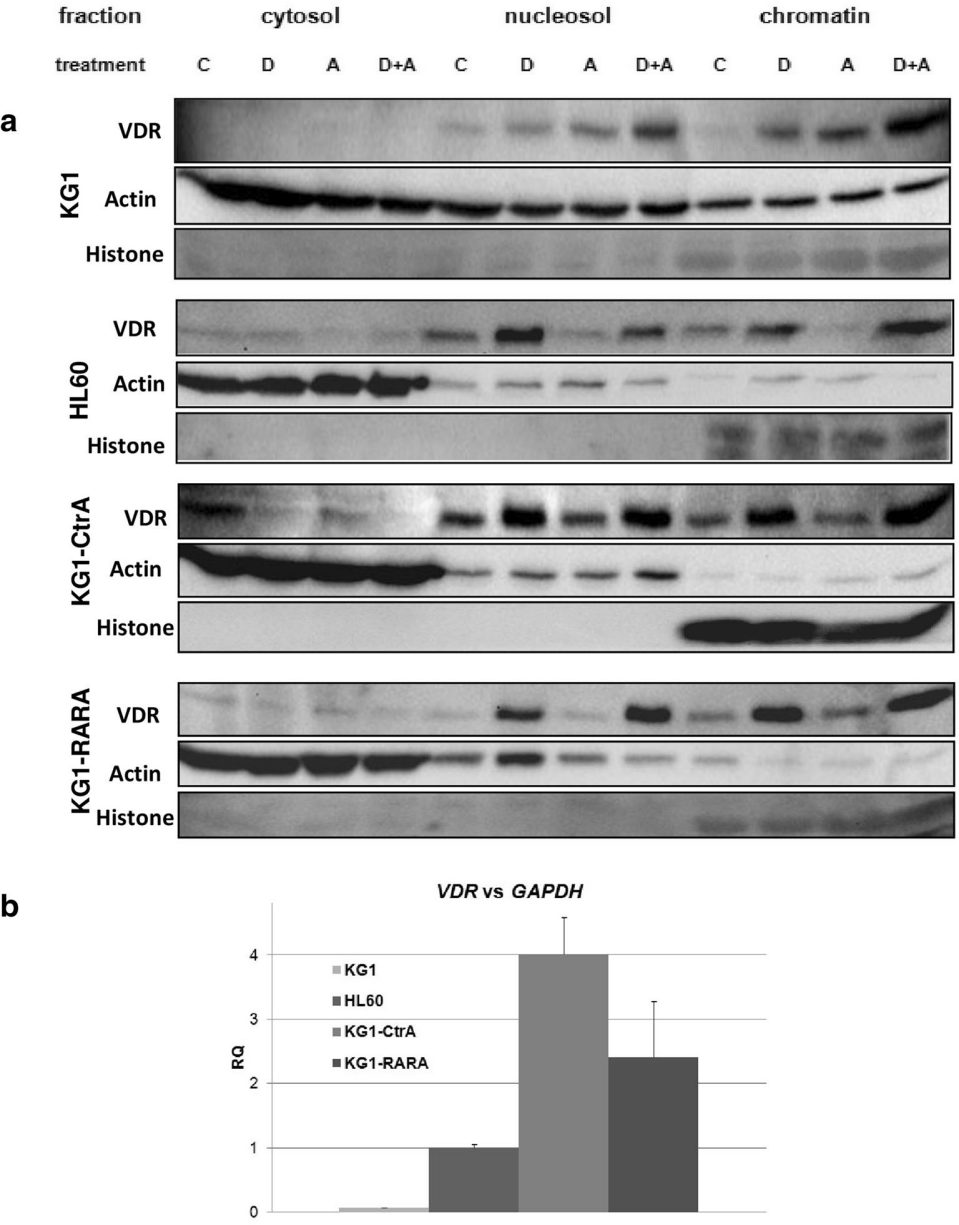
Expression of *VDR* gene and regulation of VDR and protein in AML cell lines in response to 1,25D and ATRA. KG1, HL60, KG1-CtrA and KG1-RARA cells were exposed to 10 nM 1,25D or to 500 nM ATRA or both for 72 h and then expression of VDR protein was studied (**a**). The cells were fractionated into cytosolic, nucleosolic and chromatin-bound fractions. Fractionated proteins were analyzed in western blots using anti-VDR, anti-actin and anti-Histone antibodies. The experiments were repeated from 2 (HL60) to 5 (KG1) times and representative blots are presented. *C* control, *D* 10 nM 1,25D; *A* 500 nM ATRA. The constitutive expression levels of *VDR* mRNA (**b**) in the above cell lines were measured by Real-time PCR relative to *GAPDH* expression levels. The *bar charts* show mean values of three experiments (±SEM) of relative quantity (RQ)

In order to test whether the susceptibility of KG1-CtrA and KG1-RARA cells to 1,25D-induced differentiation was mediated by VDR, we tested the levels of this protein in our cells. It has been documented that VDR in AML cells accumulates in the cell nuclei in response to 1,25D exposure [23, 24]. In KG1 cells the VDR expression level is low, but it increases after addition of ATRA [14]. Therefore, for the next experiments we exposed KG1, HL60, KG1-CtrA and KG1-RARA cells to 10 nM 1,25D, 500 nM ATRA or both for 72 h and then detected VDR in the cell cytosol, nucleosol and chromatin fractions. As presented in Fig. 2a, the level of VDR protein is low in all untreated cells, but in HL60, KG1-CtrA and in KG1-RARA it grows substantially after exposure to 1,25D, and does not change after ATRA. In KG1 cells the situation is different. In these cells 1,25D does not change the level of VDR considerably, ATRA causes a slight increase of VDR levels, but ATRA and 1,25D when added at the same time cause significant accumulation of VDR in nucleosol and in chromatin fractions. The above results show that the regulation of VDR protein level after delivery of plasmid DNA changed from the mode typical for 1,25D-resistant KG1 cells to the mode typical for 1,25D-sensitive HL60 cells. To test whether the differences in VDR protein levels between wild type KG1 cells and the transfected sublines come from protein translation, or from protein stability, the levels of *VDR* mRNA were detected in these cells, and compared to HL60 cells. As presented in Fig. 2b the constitutive expression level of *VDR* mRNA in KG1-CtrA was about 4 times higher than in HL60 cells and about 57 times higher than in KG1 cells, while in KG1-RARA cells it was about 2.4 times higher than in HL60 cells and about 35 times higher than in KG1 cells.

### VDR mRNA in HL60, KG1 and KG1-CtrA and its regulation in response to ATRA

**Fig. 3:**
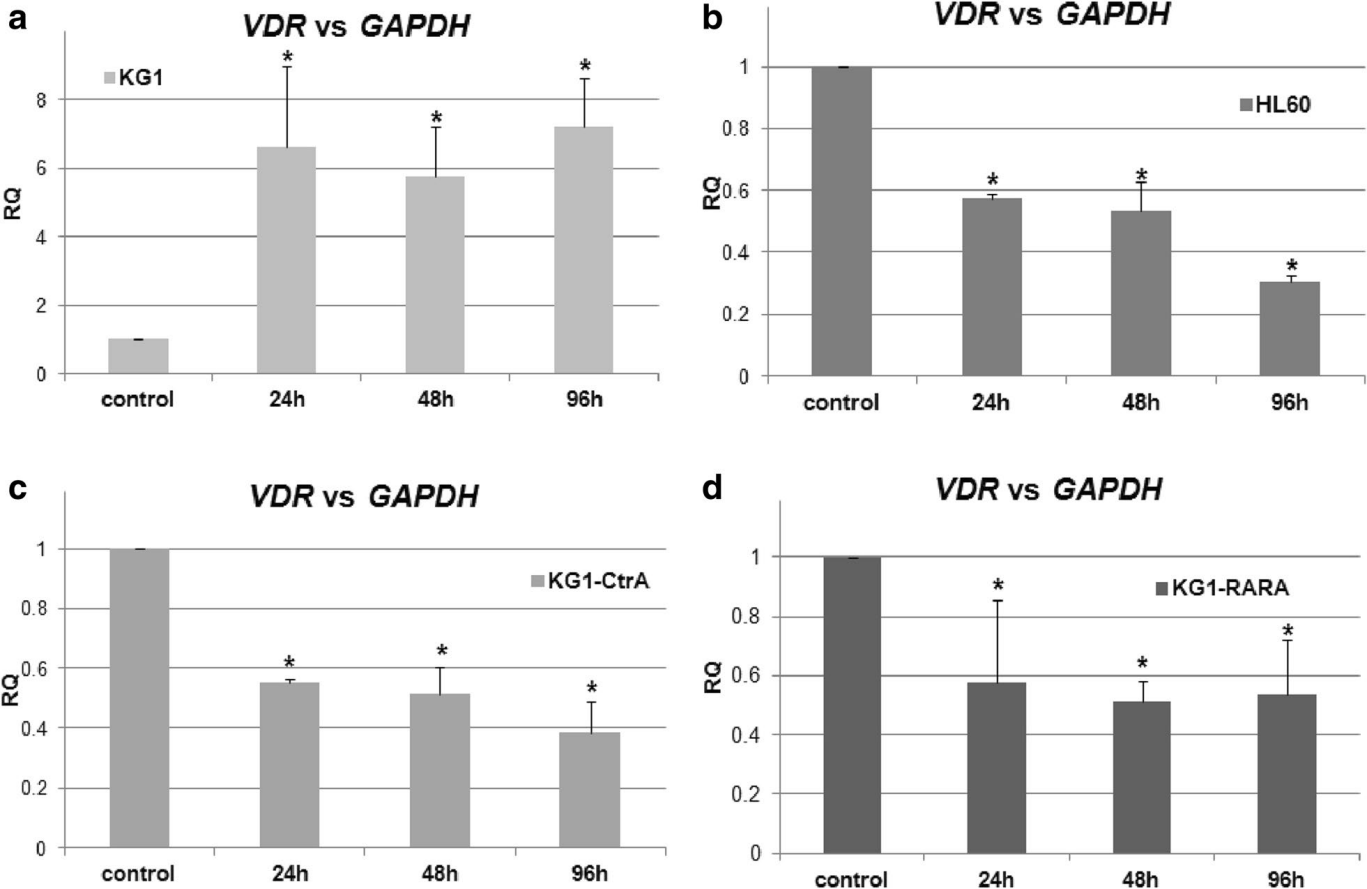
Regulation of *VDR* gene in response to ATRA in AML cell lines. KG1 (**a**), HL60 (**b**), KG1-CtrA (**c**) and KG1-RARA (**d**) cells were exposed to 1 µM ATRA for 24, 48 and 96 h and then expression of *VDR* gene was measured by Real-time PCR relative to *GAPDH* expression levels. The *bar charts* show mean values of three experiments (±SEM) of relative quantity (RQ). Values that differ significantly (p < 0.05) from those obtained for control cells are marked with *asterisks*

In our previous work we demonstrated that in HL60 and in KG1 cells *VDR* mRNA was regulated in an opposite manner in response to ATRA; in HL60 cells it was down-regulated, while in KG-1 it was up-regulated [14]. Thus, we addressed the question whether the genetic modification of KG1-CtrA and KG1-RARA cells influenced the regulation of *VDR* mRNA in response to ATRA. Thus HL60 (Fig. 3a), KG1 (Fig. 3b) KG1-CtrA (Fig. 3c) and KG1-RARA (Fig. 3d) cells were for 24, 48 and 96 h exposed to 1 μM ATRA and the expression of *VDR* mRNA was measured using Real-time PCR *versus* the expression of *GAPDH* which is stable in all these cell lines. The experiment showed that expression of *VDR* mRNA in KG1-CtrA and KG1-RARA cells was down-regulated by ATRA, similarly to HL60 cells, but unlikely wild-type KG1 cells. The fact that ATRA regulates the expression of the VDR gene in bone and mammary cells has been described in the past, but the mechanism of this regulation is not well understood [25, 26]. It should be noticed that ATRA is a non-selective ligand of the three distinct isoforms of retinoic acid receptors (RAR) α, β and γ, which occur in numerous splicing variants [27] and these isoforms may differentially contribute to the regulation of *VDR* gene expression.

### Chromatin accessibility assay in HL60, KG1 and KG1-CtrA

**Fig. 4:**
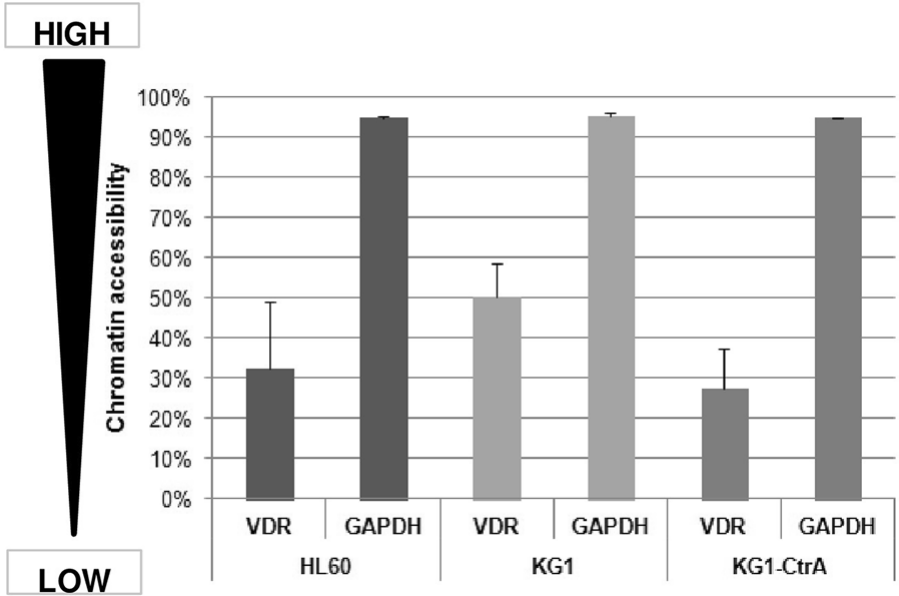
Accessibility of *VDR* and *GAPDH* proximal promoters in HL60, KG1 and KG1-CtrA. HL60, KG1 and KG1-CtrA cells were treated with nucleases in order to stimulate chromatin digestion after which chromatin was isolated, followed by Real-time PCR. The difference in C_T_ values between digested and undigested chromatin allowed for evaluation of chromatin states (% chromatin accessibility) among the cell lines. *GAPDH* was used as the control (constitutively expressed) and *RHO* was used as the reference (epigenetically silenced) gene. Data are the mean values (±SEM) of three individual experiments

We next addressed the question whether increased expression of *VDR* after delivery of plasmid DNA to KG1 cells was caused by epigenetic effects. Thus we tested if there is a difference in chromatin accessibility within these cell lines as accessibility strongly correlates with gene expression. Using the EpiQ analysis kit, the chromatin state was identified based on how accessible the DNA was to nucleases. We evaluated the accessibility of the *VDR* promoter region in HL60, KG1 and KG1-CtrA cells. In all cell lines the proximal region of the *VDR* gene showed a low degree of accessibility (in a rage of 20–65 %), which according to the test manufacturer, is considered moderately silenced when compared to that of *GAPDH*, an epigenetically “open” gene (Fig. 4). The accessibility of *VDR* was therefore identified as being in similar range of silencing in all three cell lines.

### FGFR1-FOP signaling in KG1, KG1-CtrA and KG1-RARA cells

**Fig. 5:**
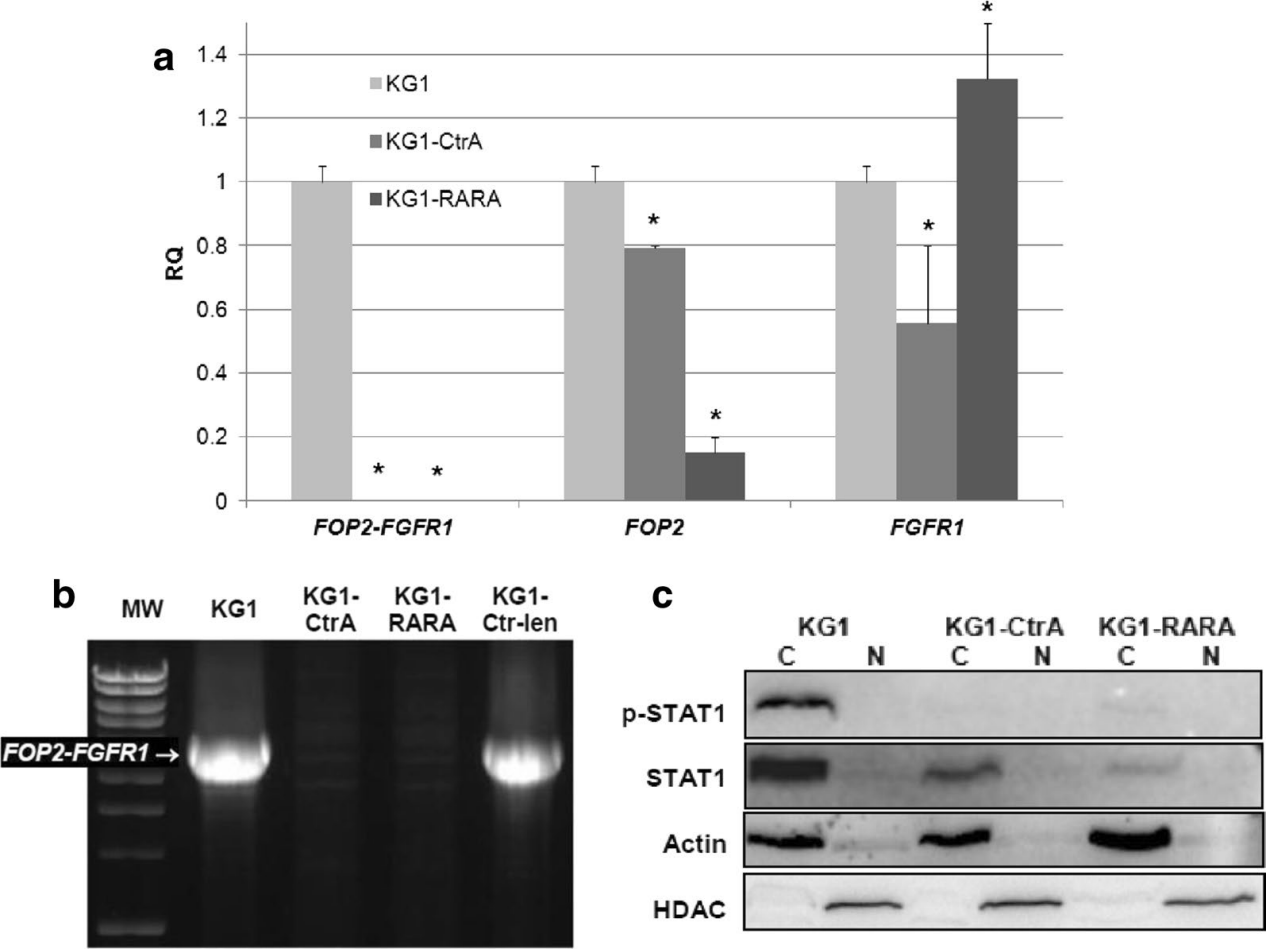
Expression of *FOP2*–*FGFR1* and presence of phospho-STAT1 in KG1, KG1-CtrA and KG1-RARA cells. The expression of wild-type *FGFR1*, wild-type *FOP2* and the fusion *FOP2*–*FGFR1* gene in KG1, KG1-CtrA and KG1-RARA cells was tested by Real-time PCR relative to *GAPDH* expression levels (**a**). The expression levels obtained for KG1 cells were calculated as 1. The *bar charts* show mean values of three experiments (±SEM) of relative quantity (RQ). Values that differ significantly (p < 0.05) from those obtained for KG1 cells are marked with *asterisks*. The region between exon 4 of *FOP2* and exon 10 of *FGFR1* within the fusion *FOP2*–*FGFR1* gene was amplified from genomic DNA obtained from KG1, KG1-CtrA, KG1-RARA and KG1-Ctr-len cells (**b**). The PCR products were separated in 1 % agarose gel and visualized with ethidium bromide under UV light. Total cell lysates from KG1, KG1-CtrA and KG1-RARA cells were analyzed in western blots for the presence of active STAT1 and total STAT1 level (**c**). Actin content was tested as a control of equal loading and transfer of proteins. The experiment was repeated two times, each in duplicate, and illustrative blot is presented

In further search for a molecular mechanism of the restored susceptibility to 1,25D in transfected KG1 sublines, we addressed the oncogene which drives malignant transformation in KG1 cells. KG1 cells have been reported to express the wild-type *FGFR1*, wild-type *FOP2* and the fusion *FOP2*–*FGFR1* gene and protein [2]. Thus we tested the levels of mRNA for *FOP2*–*FGFR1* fusion gene, *FOP2* and *FGFR1* in KG1, KG1-CtrA and KG1-RARA cells, normalized to *GAPDH* expression levels using Real-time PCR. We observed that the expression of the fusion *FOP2*–*FGFR1* gene which is high in KG1, was undetectable in KG1-CtrA and KG1-RARA cells (Fig. 5a). Next, the question of fusion gene integrity in transfected cells was addressed. The whole sequence of the fusion *FOP2*–*FGFR1* gene is not available, but the region between exon 4 of *FOP2* and exon 10 of *FGFR1* has been studied. This region contained the elements from intron 4–5 of *FOP2*, the inverted and truncated exon 9, and intron 9–10 of *FGFR1* [28]. We decided to amplify the above region from the genomic DNA isolated from wild-type KG1 cells as well as from KG1-CtrA and KG1-RARA cells. As presented in Fig. 5b, in KG1 and in KG1-Ctr-len cells the expected product of about 5 kb pairs was present, while in KG1-CtrA and KG1-RARA cells it was absent, showing that transfection with plasmid DNA disrupted the fusion gene integrity.

As it has been presented in the past, there is a constitutive activation of STAT1 transcription factor in KG1 cells [3]. In our experiments we tested, if this transcription factor is also constitutively active in KG1-CtrA and KG1-RARA cells. We tested the presence of Tyr-701 phospho-STAT1 in these cells, relative to the total amount of STAT1 and to the actin content. The results presented in Fig. 5c document constitutive activation of STAT1 in KG1 cells, but neither in KG1-CtrA nor in KG1-RARA cells. However, not only the levels of phosphorylated STAT1 were higher in wild type, than in transfected cells. The total amount of STAT1 was also higher in KG1 than in both transfected sublines, when compared to the actin content.

### IFN stimulated genes in HL60, KG1, KG1-CtrA and KG1-RARA cells

**Fig. 6:**
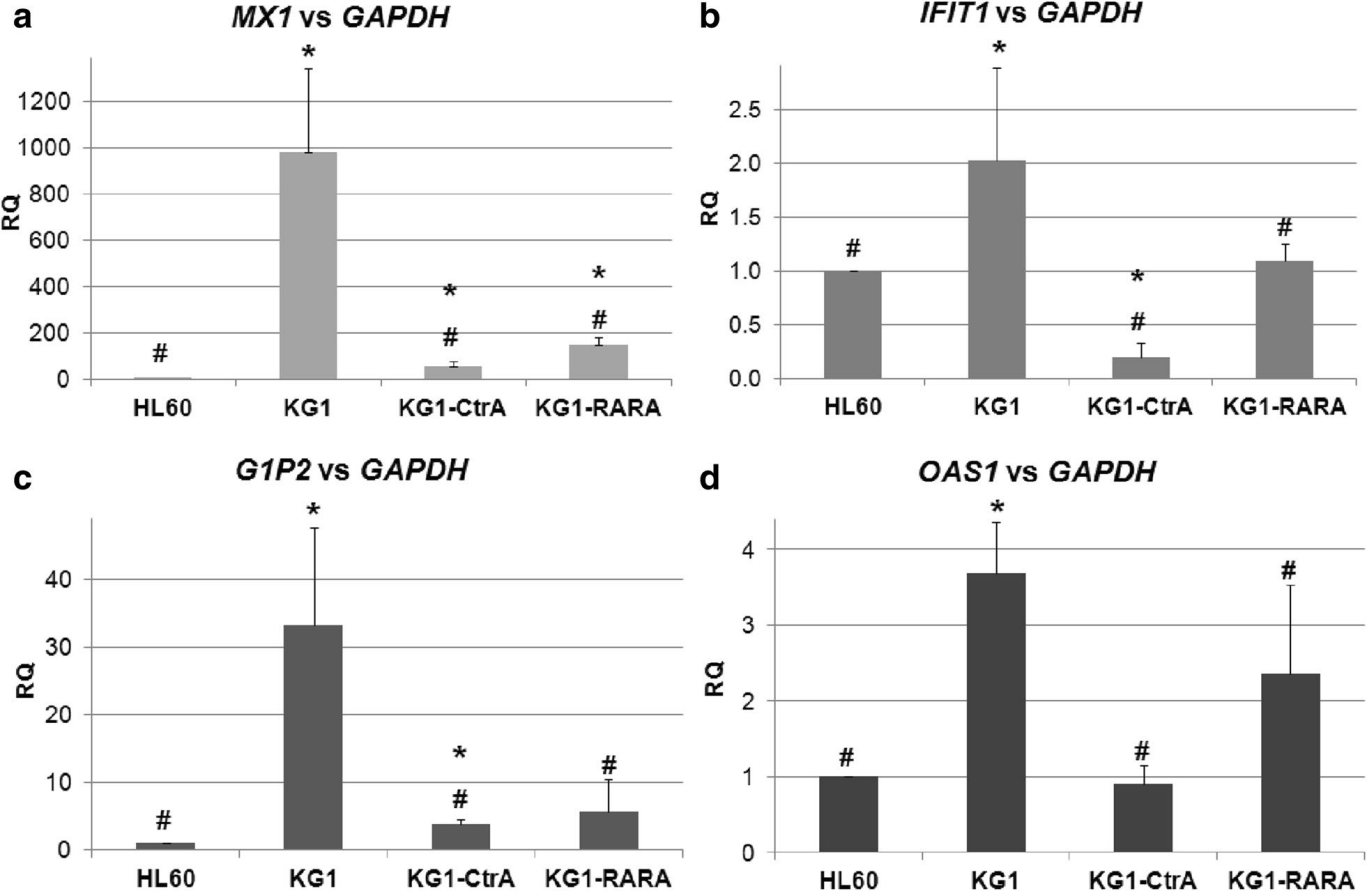
Expression of ISGs in AML cell lines. Transcription levels of *MX1* (**a**), IFIT1 (**b**), *G1P2* (**c**) and *OAS1* (**d**) were measured in HL60, KG1, KG1-CtrA and KG1-RARA cells by Real-time PCR relative to *GAPDH* expression levels. The expression levels obtained for HL60 cells were calculated as 1. The *bar charts* show mean values of three experiments (±SEM) of relative quantity (RQ). Values that differ significantly (p < 0.01) from those obtained for HL60 cells are marked with *asterisks*, while the values that differ significantly (p < 0.02) from those obtained for KG1 cells are marked by *hash*

Since STAT1 was constitutively active in KG1 cells, but no longer in KG1-CtrA and KG1-RARA cells, we were interested in downstream events in these cell lines. Transcription factors from STAT family are activated predominantly by IFNs, so we decided to test ISGs expression. For that purpose we used commercially available IFNr qRT-Primers kit, which allows to quantify the expression of five well characterized ISGs, which we tested in HL60, KG1, KG1-CtrA and KG1-RARA cells. As presented in Fig. 6 expression levels of *OAS1*, *MX1*, *G1P2* and *IFIT1* were significantly higher in KG1 cells than in KG1-CtrA, KG1-RARA and HL60. The only ISG tested, which wasn’t upregulated in KG1 cells was *IFNB* gene (not shown). It is noteworthy, that in normal cells signal transduction from IFNs causes not only phosphorylation and activation of STAT1 transcription factor, but also upregulates *STAT1* gene expression [29]. This is why *STAT1* is in some publications included into the group of ISGs [30]. Therefore, high STAT1 protein content in KG1 cells (presented in Fig. 5b) most probably might be attributed to the constitutive activation of IFN signaling in these cells.

### Differentiation of KG1, KG1-CtrA and KG1-RARA cells after externally added IFNs

**Fig. 7:**
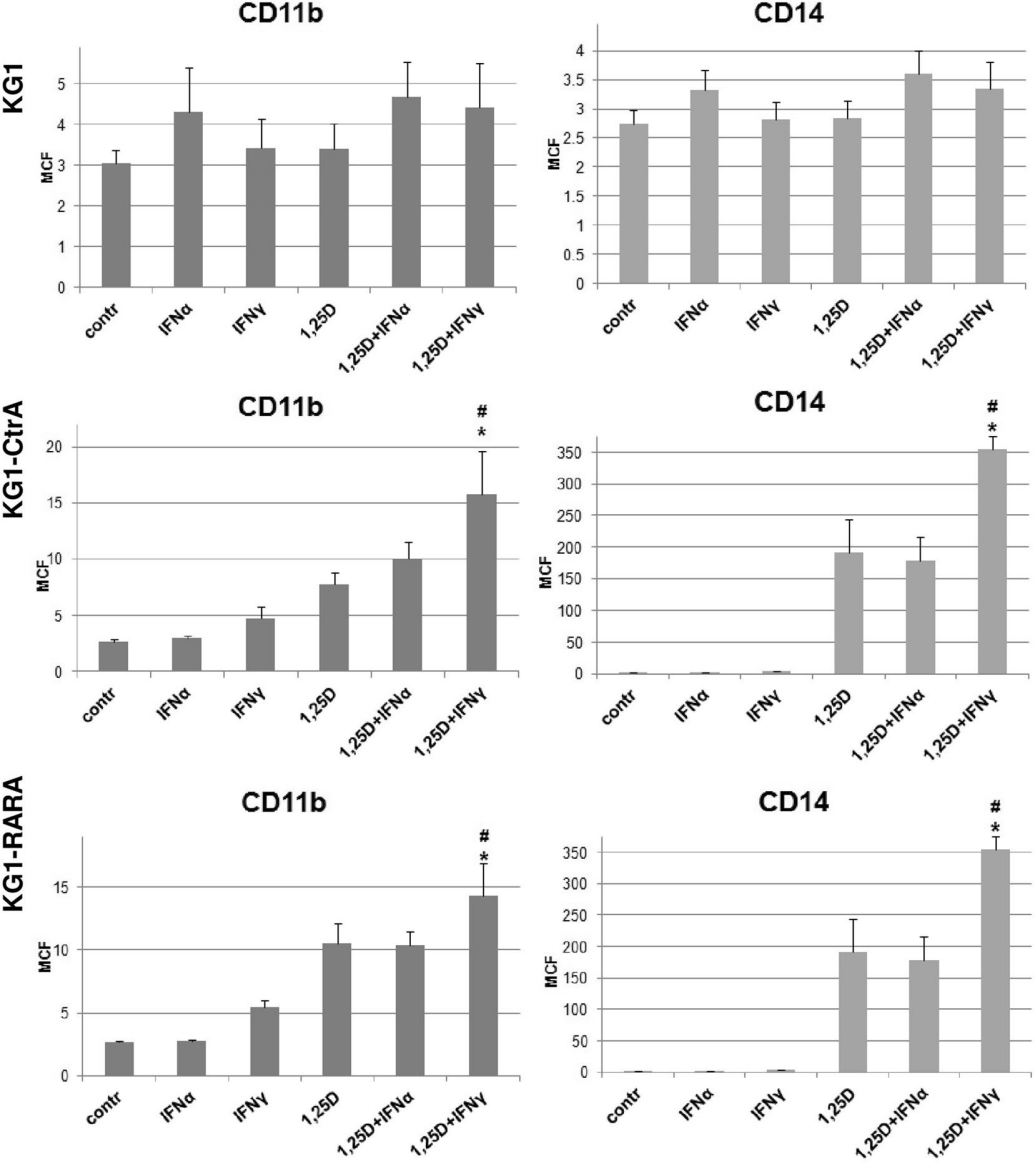
Differentiation of KG1, KG1-CtrA and KG1-RARA cells in response to IFNs and 1,25D. KG1, KG1-CtrA and KG1-RARA cells were exposed to 10 ng/ml of IFNα, 10 ng/ml of IFNγ with or without 10 nM 1,25D for 96 h and then the expression of CD11b and CD14 differentiation markers was detected using flow cytometry. Experiments were repeated 3 (KG1 and KG1-RARA) or 4 (KG1-CtrA) times. The bar charts show the density of CD11b and CD14 antigens on the cell surface, expressed as mean channel of fluorescence (MCF) values (±SEM). Values that differ significantly (p < 0.05) from those obtained for 1,25D treated cells are marked with *asterisks*, while the values that differ significantly (p < 0.01) from those obtained for IFNγ-treated cells are marked by *hash*

As it has been reported in the past, IFNα and IFNγ augment the differentiation of leukemic cells induced by other factors [31]. For example, it has been shown that blast cells from patients with chronic myeloid leukemia when cultured with IFNα and granulocyte–macrophage colony stimulating factor, developed a morphology characteristic for dendritic cells [32]. Since KG1 cells, where INF-like signaling was constitutively switched on, did not resemble antigen presenting cells, we have addressed the question what would be the effect of externally added IFNs. We have tested various concentrations of IFNα and IFNγ, and we found that 10 ng/ml dose was the highest, that did not cause toxicity after 96 h exposure time. KG1, KG1-CtrA and KG1-RARA cells were thus treated with IFNα or IFNγ with or without 10 nM 1,25D for 96 h, and then the expression of CD11b and CD14 cell surface markers was tested in flow cytometry. As presented in Fig. 7, there was no effect of IFNs towards KG1 cells, where expression of CD11b and CD14 was similar to the control values in all treated samples. On the contrary, in KG1-CtrA and in KG1-RARA cells, IFNγ was able to significantly increase levels of CD11b and CD14 molecules at the cell surface and it acted in an additive manner with 1,25D.

### Differentiation of KG1 after externally added curcumin

**Fig. 8:**
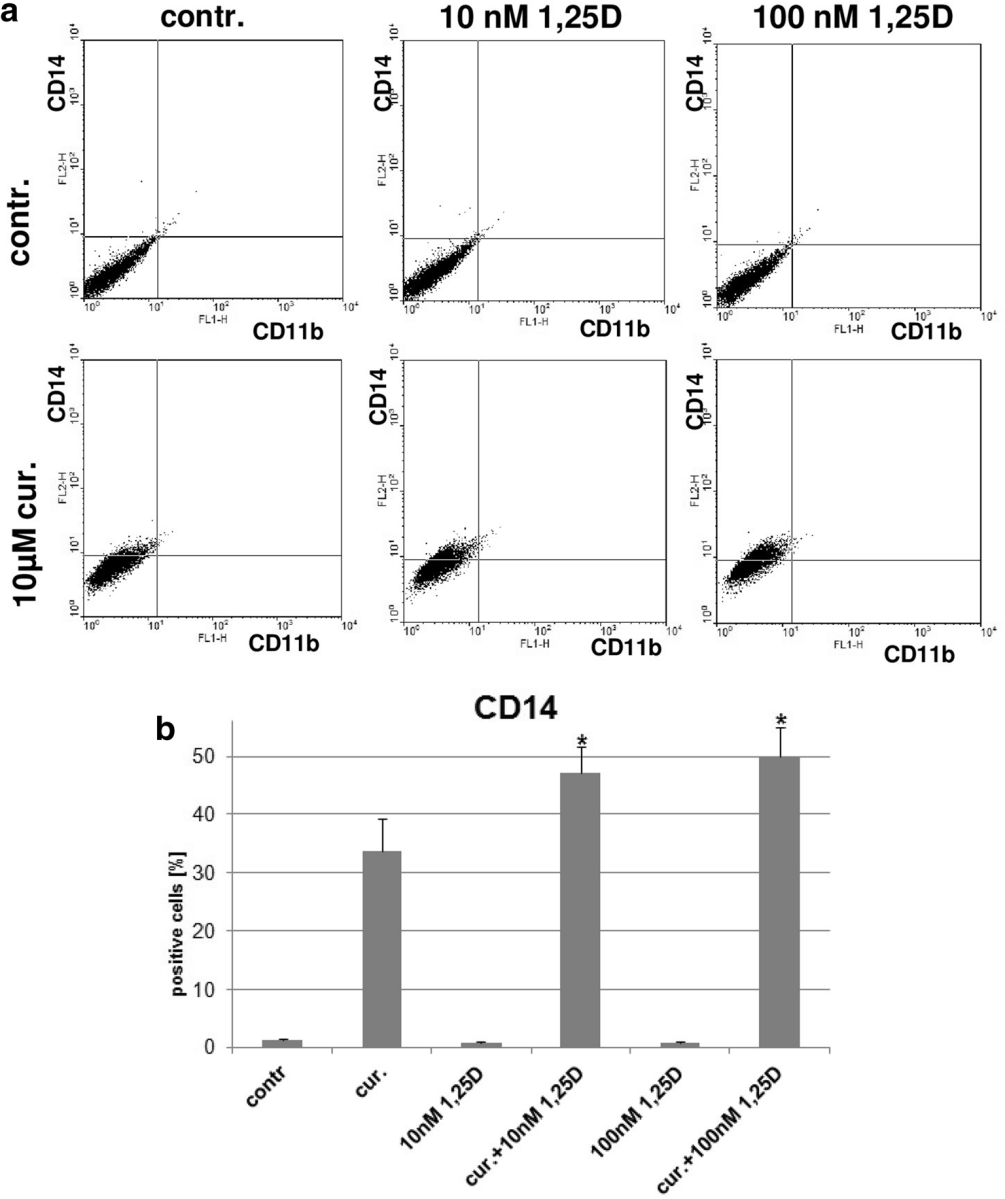
Differentiation of KG1 cells in response to curcumin and 1,25D. KG1 cells were exposed to 10 µM curcumin with or without 1,25D (10 or 100 nM) for 96 h and then the expression of CD11b and CD14 differentiation markers was detected using flow cytometry. Addition of 1,25D at both concentrations significantly enhanced the expression of CD14 in KG1 cells when compared to curcumin-treated cells. The experiments were repeated 5 times and illustrative dot-blots are presented in **a**, while the mean percentages of positive cells (±SEM) are presented in **b**. The samples that differ significantly from the curcumin-treated cells are marked with *asterisk* (p < 0.02)

RNAse L is a latent enzyme, expressed in nearly every mammalian cell type. In its latent state it is inhibited by an intrinsic Ribonuclease L Inhibitor and it is activated in response to IFNs. The only external and cell permeable RNAse L inhibitor documented until now is curcumin, which in vitro inhibits the enzyme activity at 5–10 µM concentrations [33]. Therefore, we decided to test, if the addition of curcumin would restore 1,25D-induced differentiation in wild type KG1 cells. It appeared that 10 µM curcumin itself is quite efficient differentiation-inducing factor in KG1 cells, however, the differentiation is limited to CD14 cell surface marker with no increase in CD11b. Curcumin and 1,25D added simultaneously to KG1 cells produced a synergistic effect of CD14 expression (Fig. 8). This suggests that in part the resistance of KG1 cells to 1,25D was caused by RNAse L activation.

## Discussion

Delivery of DNA into cancer cells in vitro and in vivo has become a standard protocol worldwide. It has been believed that delivery of control plasmid DNA has no significant effect to the cells, however some data were published which show that this is not always the truth. It has been shown that in some tumor cell lines delivery of control plasmid DNA caused significant increase in the expression of following ISGs: *IRF7*, *STAT1*, *MIG*, *MICA* and *ITGAL* [30]. The phenomenon of activation of IFN signaling in transfected cells was attributed predominantly to delivery of short interfering (si) RNAs to the cells [34]. In this paper we show that plasmid DNA delivered to acute myeloid leukemia cells may integrate into genomic DNA and disrupt *FOP2*–*FGFR1* fusion gene. Our results show that this “side effect” of DNA delivery might have positive influence towards cell phenotype.

The above phenomenon has encouraged us to address the question of low *VDR* expression level in KG1 cells. Disruption of *FOP2*–*FGFR1* fusion gene unexpectedly restored sensitivity of KG1 cells to 1,25D. This was caused by increased levels of *VDR* mRNA, followed by translation of VDR protein. VDR protein in genetically modified KG1-CtrA and KG1-RARA cells was transcriptionally functional and these cells resembled 1,25D-sensitive HL60 cells. In transfected KG1 sublines 1,25D induced expression of CD14 cell surface marker, which is a characteristic feature of mature monocytes and necessary for phagocytosis [35]. Moreover, the regulation of *VDR* expression by ATRA, which in KG1 cells is opposite from that in HL60 cells, showed to be identical in both HL60, KG1-CtrA and KG1-RARA cells. We then hypothesized that the transcription of VDR gene was silenced in KG1 cells in comparison with KG1-CtrA or HL60 cells. Since the epigenetic mechanisms of gene silencing are variable, we took advantage of EPIQ chromatin analysis test. The results of this test show the accessibility of chromatin in the promoter region of studied gene, regardless the epigenetic modifications. These results showed that promoter region of *VDR* has a moderate degree of accessibility within all HL60, KG1 and KG1-CtrA cell lines, thus epigenetic gene silencing was not the reason of various *VDR* expression levels in HL60, KG1 and KG1-CtrA cells.

Subsequently, we looked at the aberrant signal transduction in KG1 cells. It was reported before that transcription factor STAT1 is constitutively active in KG1 [3]. Indeed, we have found high levels of STAT1 protein, and its constitutive activation in KG1 cells, but not longer in KG1-CtrA and in KG1-RARA cells. In response to disruption of *FOP2*–*FGFR1*, STAT1 signal transduction became switched off, which pointed out that constitutively active fusion FOP2–FGFR1 kinase is an upstream activator of this pathway.

Among the ISG genes constitutively activated in KG1 cells were the ones that code for OAS proteins, which in turn activate RNase L, highly regulated, latent endoribonuclease [36]. Since *OAS* genes were overexpressed in KG1, but neither in HL60, KG1-CtrA nor in KG1-RARA cells, the degradation of mRNA could be one of the reasons of VDR low level. In order to verify whether RNAse L activation contributes to the resistance of KG1 cells to 1,25D, we used curcumin, which has been reported as the only cell permeable RNAse L inhibitor. Our experiments revealed, that curcumin itself is a differentiation-inducing factor towards KG1 cells, and in combination with 1,25D it had synergistic pro-differentiation effect. It should be noted however, that degradation of *VDR* RNA may in part contribute to resistance of KG1 cells to 1,25D, but it is not the only reason.

## Conclusions

In summary, we show in this paper that delivery of plasmid DNA to the cells may disrupt fusion gene *FOP2*–*FGFR1* which occurs in a disease entity called 8p11 myeloproliferative syndrome. Whether this is limited to the cell line or present also in blast cells from patients needs to be studied in future, even though this disease is very rare. More importantly, inhibition of the FOP2–FGFR1 signal transduction pathway restored sensitivity of the cells with fusion kinase to 1,25D-induced cell differentiation. We suppose that this finding needs to be explored in more detail as it can be important for therapeutic purposes.

## Methods

### Cell lines and cultures

HL60 cells were a from a local cell bank at the Institute of Immunology and Experimental Therapy in Wroclaw (Poland), while KG1 cells were purchased from the German Resource Center for Biological Material (DSMZ GmbH, Braunschweig, Germany). The cells were grown in RPMI-1640 medium with 10 % fetal bovine serum, 100 units/ml penicillin and 100 µg/ml streptomycin (Sigma, St Louis, MO) and kept at standard cell culture conditions.

### Chemicals and antibodies

1,25D was purchased from Cayman Europe (Tallinn, Estonia), ATRA and curcumin were from Sigma. The compounds were dissolved in an absolute ethanol to 1000 × final concentrations, and subsequently diluted in the culture medium to the required concentration. IFNα (cat. no. 11343506), IFNγ (cat. no. 11343536) and antibodies CD11b-FITC (cat. no. 21279113) and CD14-PE (cat. no. 21270144), as well as appropriately labeled isotype controls were from ImmunoTools (Friesoythe, Germany). Mouse monoclonal anti-VDR (sc-13133), anti-Stat1 p84/p91 (sc-464) and anti-p-STAT1 (sc-8394), rabbit polyclonal anti-actin (sc-1616), anti-HDAC1 (sc-7872) and anti-Histone H1 (sc-10806) were from Santa Cruz Biotechnology Inc. (Santa Cruz Biotechnology Inc., CA). Goat anti-rabbit IgG, anti-mouse IgG conjugated to peroxidase, anti-mouse conjugated to biotin and streptavidin conjugated to peroxidase were from Jackson ImmunoResearch (West Grove, PA).

### Transfection reagents and procedure

Electrotransfection by Neon^®^ Transfection System (Invitrogen™, Carlsbad, CA) was performed as before [22] using control shRNA plasmid-A (sc-108060) or *RARA* shRNA plasmid (sc-29465-SH; both Santa Cruz). In order to obtain additional control cells, KG1 cells were seeded on 24-well plates (2 × 10^4^ cells per well) and after 24 h the cells were infected with 20 μl of lentiviral particles containing scrambled shRNA sequences (sc-108080; Santa Cruz) in medium containing 1 μg/ml polybrene (Santa Cruz) for 8 h. The medium was changed and the cells were grown for 2 more days. After transfection the cells were grown in a medium supplemented with 1 µg/ml puromycin (Santa Cruz). Medium and selective antibiotic were changed every 2 days and puromycin non-resistant cells were cleared from the culture.

### Flow cytometry

The expression of cell surface markers of differentiation was determined by flow cytometry. The cells were incubated with 1,25D ± IFNs or curcumin for 96 h, then washed and stained with 1 µl of fluorescently labeled antibody (or the appropriate control immunoglobulins) for 1 h on ice. Next, they were washed with ice-cold PBS and suspended in 0.5 ml of PBS supplemented with 0.1 % BSA prior to analysis on FACS Calibur flow cytometer (Becton–Dickinson, San Jose, CA). Experiments were repeated at least three times. The acquisition parameters were set for an isotype control. Data analysis was performed with use of WinMDI 2.8 software (freeware by Joseph Trotter).

### Real-time PCR

Isolation of total RNA, reverse transcription into cDNA and Real-time PCR reactions were performed as published before [14], using CFX Real-time PCR System (Bio-Rad Laboratories Inc., CA). The sequences of *VDR* and *GAPDH* primers together with reaction conditions were described previously [37]. The *FOP2*–*FGFR1*, *FOP2* and *FGFR1* primers were as published before [2]. The interferon response was evaluated by IFNr qRT-Primers (Invivogen) which allow to quantify the mRNA expression of well characterized IFN-induced genes: *IFNB*, *OAS1*, *MX1*, *G1P2*, *IFIT1*. Relative quantification (RQ) of gene expression was analyzed with ∆∆Cq method using *GAPDH* as the endogenous control. Experiments were repeated at least three times.

### Western blotting

In order to prepare cytosolic, nucleosolic and chromatin fractions 5 × 10^6^ cells/sample (equivalent of 15 μl packed cell volume) were washed with PBS and lysed using either Pierce Subcellular Protein Fractionation Kit or NE-PER Nuclear and Cytoplasmic Extraction Reagents (both from Thermo Fisher Scientific Inc., Worcester, MA) according to the user’s manual. Obtained lysates were denatured by adding 5× sample buffer and boiling for 5 min. For western blotting 25 μl of lysates were separated on 10 % SDS-PAGE gels and transferred to PVDF membranes. The membranes were then dried, and incubated sequentially with primary (3 h) and a horseradish peroxidase-conjugated secondary antibody (1 h) at room temperature. In case of STAT1 detection biotin-conjugated secondary antibody and peroxidase-conjugated streptavidin were used. The protein bands were visualized with a chemiluminescence (Santa Cruz). Then the membranes were stripped, dried again and probed with subsequent antibodies. These experiments were repeated 2–5 times.

### Chromatin analysis

Digestion of chromatin was carried out using EpiQ chromatin Analysis Kit according to manufacturer’s guidelines (Bio-Rad). All cells were viable and actively growing in culture at the time of experiment, approximately 2.5 × 10^5^ cells per sample were harvested. Cells were pelleted and resuspended in 100 µl of chromatin buffer. Digested (D) samples were treated with 2 µl of EpiQ nuclease, undigested (U) samples were not treated with nuclease. Both D and U samples were incubated at 37 °C for 1 h. Stop buffer was added to the samples for 10 min at 37 °C to stop chromatin digestion and thereafter the genomic DNA was extracted and purified, chromatin accessibility was then assessed by real-time quantitative PCR using CFX Real-time PCR system. For each cell type three digested samples and three undigested samples were analyzed using EpiQ chromatin Kit Data Analysis Tool (http://www.bio-rad.com/epiq) and normalized against the *RHO* (Rhodopsin) gene as a negative reference to a closed chromatin structure. The *GAPDH* gene was a positive reference to an open chromatin structure. Primers to analyze the proximal promoter of the human *VDR* gene were designed as recommended by the manufacturer using Primer3 software. The sequences for *VDR* were forward: 5′-GGCTGAAGCGGGTATCCGCACCTAT-3′, and reverse: 5′-TTTGACAAGCAGAGACAGCCCAGCA-3′. Experiments were repeated three times.

### PCR reaction

Genomic DNA from 5 × 10^6^ of cells was isolated using GenElute™ Mammalian Genomic DNA Miniprep Kit (Sigma). *FOP2* forward (AGATGATCCGGGTATAATAA) and *FGFR1* reverse (AGAAGAACCCCAGAGTTCAT) primers were used to amplify the ~ 5 kb genomic fusion sequence [28]. A PCR reaction was performed with 1 μl of Marathon DNA polymerase (A&A Biotechnology, Gdansk, Poland), 250 μM of each dNTP, 200 ng of each primer and 500 ng of genomic DNA in 50 μl of reaction mixture. The PCR reaction conditions were according to the polymerase protocol with annealing temperature of 53 °C, 5 min of elongation step and with 35 cycles. PCR products were visualized on a 1 % agarose gel stained with ethidium bromide and HyperLadder™ 1 kb and 25 bp (Bioline, London, UK).

### Statistical analysis

The Student’s *t* test for independent samples was used to analyze the results obtained in experiments (Excel, Microsoft Office).

## Abbreviations

1,25D: 1,25-dihydroxyvitamin D_3_
AML: acute myeloid leukemia
ATRA: *all*-trans retinoic acid
FGFR1: fibroblast growth factor receptor 1
FLT3: Fms-related tyrosine kinase 3
FOP2: FGFR1 oncogene partner 2
IFN: interferon
ISGs: IFN stimulated genes
MCF: mean channel of fluorescence
RARA: retinoic acid receptor α gene
RNAse L: ribonuclease L
RQ: relative quantity
sh: short hairpin
STAT: signal transducer and activator of transcription
VDR: vitamin D receptor
